# Nursing Affairs in Ireland

**Published:** 1917-10-20

**Authors:** 


### NURSING AFFAIRS IN IRELAND.

### The Red Cross Pageant.

DUBLIN is busy this week preparing for Our Day, and by the time this issue of THE HOSPITAL is in the hands of its readers a grand Red Cross Pageant will have paraded the principal thoroughfares of the city. The organiser is Mr. Charles Manners, of opera fame, and the theme on which the pageant is based is the evolution of nursing. Mr. Manners begins with Chaos, or, otherwise, with the time when nursing on the battlefield was unknown. Following this depressing period there comes, on horseback, a fully-equipped Knight of the Order of St. John of Jerusalem, clad in armour and bearing a sword—for it must not be forgotten that the Knights of St. John, having established a hospital at Jerusalem for Latin pilgrims, acted in the dual capacity of hospital attendants and militant knights. The modern embodiment, of course, of the ancient Order is the first-aid man of the St. John Ambulance Brigade. Following immediately after this distinguished figure comes the Alchemist, the magician, man of science, and sometimes the charlatan of mediæval England. Then comes the immortal Florence Nightingale, bearing aloft her lamp of love; and, to make the picture more real, there will accompany her, in uniform, some Crimean veterans able to bear personal testimony to the miracle that she wrought in her hospital at Scutari. Next in processional order comes Lord Lister, with his antiseptic equipment. The remainder of the procession is to consist of present-day Red Cross Hospital accoutrements—men and women carrying banners and stretchers, military cookers with their cooks, an operating table, *x*-ray apparatus—the whole being accompanied by four military bands. It was hoped that the service of the White Ensign would have been represented, for there is a fairly large naval contingent constantly at Kingstown, but the demands of the Admiralty are at the moment strenuous, and it has to be said with regret that there will not be a single bluejacket present. Lady Arnott, who is chairman of the Pageant Committee, hopes to be able to procure the attendance of an airship, but as I write this interesting feature is in the lap of the gods. The pageant promises to be interesting as well as instructive, and will help the man in the street to realise something concerning the man in the trench, which is the essence of Red Cross propaganda.

The organiser-in-chief of Our Day in the three southern provinces of Ireland is Doctor John Lumsden, who is about to proceed on active service to France. He is the head of the St. John Ambulance Brigade in Ireland, and is to be entertained to dinner at the Gresham Hotel on Saturday next, prior to his departure. Dr. Lumsden has laboured in season and out of season, since the outbreak of the war, in the Red Cross cause. Nobody knows, and nobody probably will ever know, how much of his life Dr. Lumsden has devoted to the cause of the Red Cross. His is the hidden hand that inspires, directs, and guides. He is held in the highest esteem by members of the Brigade of all ranks, who hope that even the strenuous duties of the field will be a relaxation from the constant worrying care of his multifarious duties at home.

### An Irish Midwives Bill.

It is expected that an Irish Midwives Bill will be presented to Parliament during the current month. It will be a Government measure, and, doubtless, it will become law, as there is not likely to be any opposition, and the need for legislation is pressing. The provisions of the Bill are so far secret, and nothing is likely to be known until it is read in Parliament. There are, however, fears that, as I believe is the case in Scotland, midwives who qualify for an Irish C.M.B. diploma will not be eligible for employment in England, although English midwives may practise here, and consequently it is intended to enlist the services of Irish M.P.s to overcome any such disability. Now that the bonds of Empire are being closer drawn it is surely desirable that the Imperial spirit of the College of Nursing should inspire all nursing legislation. The Irish midwives want a Bill that will confer upon them powers to practise in Great Britain and the Colonies. Perhaps the Central Midwives Board in London will display an interest in bringing this about.

### College Propaganda.

Concerning the coming public meeting of nurses to be held under the auspices of the College of Nursing, the date of which had to be altered and is not yet fixed, the subject of finance ought to be handled with great frankness. It is being sedulously and insidiously disseminated by College opponents amongst Irish nurses that their guineas are to be collected from them and spent in England. This utterly untrue suggestion finds ready listeners, especially at the moment when violent political factions are at work, and, in my opinion at all events, it is far better to face the music if there is music to be faced. Nurses have only to reflect, and to use a little common-sense, to realise that Ireland is a barren country so far as the nursing profession is concerned, and if the College ever becomes a flourishing establishment in Ireland, even in the most favourable conditions, headquarters cannot hope to derive any profit from Irish subscriptions. As a matter of fact, the Irish Nurses' Association in its best days was a poverty-stricken institution, and this year the pittance paid to the secretary had to be reduced.

### "An Absurd Procedure."

The doctrine of earmarking Irish moneys for expenditure in Ireland finds support in many quarters. Even Red Cross contributions are occasionally so earmarked—a most absurd procedure, seeing that the armies of the Red Cross are not at home, but encamped behind the trenches where British soldiers are shedding their life's blood. Ignorance—and pardonable ignorance at that—is responsible for the readiness with which slander and false suggestions are scattered broadcast. In the circumstances it is eminently desirable that the air should be cleared. This can only be accomplished by a clear, outspoken statement of facts and figures which will bear analysis and irrefutable demonstration. When the rank and file realise that the object of the College of Nursing is the benefit and the unification of the profession, and not a money-making concern, the atmosphere of suspicion will be dissipated. Just now there are several contending factions, each out for recruits. The rank and file have only one end to achieve, and ultimately, if not immediately, they will rally round the standard that stands for progress, for enlightenment, and for betterment. IERNE.

